# Mechanism and Regulation of Tea Saponin Extraction from *C. oleifera* Seed Meal in Subcritical Water

**DOI:** 10.3390/foods14111849

**Published:** 2025-05-22

**Authors:** Aifeng Niu, Chengming Wang, Fangrong Liu, Guowei Ling, Yu Wang, Shilin Liu, Xizhou Hu

**Affiliations:** 1 College of Food Science and Technology, Huazhong Agricultural University, Wuhan 430070, China; niuaifeng@webmail.hzau.edu.cn (A.N.); l15884925210@webmail.hzau.edu.cn (F.L.); lingguowei@webmail.hzau.edu.cn (G.L.); wy0912@webmail.hzau.edu.cn (Y.W.); slliu2013@mail.hzau.edu.cn (S.L.); 2 Key Laboratory of Environment Correlative Dietology, Huazhong Agricultural University, Ministry of Education, Wuhan 430070, China; 3 Institute of Agricultural Quality Standards and Testing Technology Research, Hubei Academy of Agricultural Sciences, Wuhan 430064, China; huxizhou@163.com

**Keywords:** tea saponins, proteins, reducing sugars, Maillard reaction, subcritical water

## Abstract

Tea saponins are excellent natural surfactants, and previous studies on their extraction from *C. oleifera* seed meals in subcritical water have mainly focused on the optimization of external extraction conditions. In order to achieve the efficient extraction of tea saponins in subcritical water, this study explores the influence of the composition-internal factors on the extraction rate of tea saponins. In this study, the composition of three *C. oleifera* seed meals purchased from Hubei, Hunan and Guizhou province and extraction rates of tea saponins, dissolution rates of reducing sugars and proteins from these *C. oleifera* seed meals were compared, and the results showed that reducing sugars and proteins were intrinsic components affecting extraction rates of tea saponins in subcritical water. The simulation system involving tea saponins, whey protein isolate (WPI), and glucose in subcritical water showed that WPI reduced the content of tea saponins through the Maillard reaction, and glucose inhibited the participation of tea saponins in the Maillard reaction. The above mechanism was verified using alkaline protease, which changed the content of reducing sugars and proteins in the *C. oleifera* seed meal purchased from Hubei province, and provided guidance for achieving the efficient extraction of tea saponins.

## 1. Introduction

Tea saponins are a mixture of oleanane-type pentacyclic triterpenoid saponins, whose basic structure consists of three parts: saponins, glycosides, and organic acids [1], and are distributed in the flowers, leaves, and seeds of *C. oleifera* [2]. Tea saponins are excellent natural non-ionic surfactants, which are widely used in the food, chemical, and medicine industries [3]. The *C. oleifera* seed meal is a by-product of oil extraction from *C. oleifera* seeds. In China, the annual output of *C. oleifera* seed meal is relatively high, which can reach 690,000 metric tons [4]. And the *C. oleifera* seed meal is an excellent source of tea saponins [5]. The extraction of tea saponins from *C. oleifera* seed meals can also improve the utilization value of *C. oleifera* seed meals.

At present, the main extraction methods for tea saponins from *C. oleifera* seed meals are hot water extraction [6], ethanol extraction [7], ultrasonic and microwave-assisted extraction [8,9], and new auxiliary extraction methods such as subcritical water. Subcritical water refers to water at temperatures ranging from 100 °C to 374 °C. The polarity of subcritical water is regulated by varying the temperature and pressure to realize the selective extraction of substances with different polarities [10]. Moreover, subcritical water extraction uses only water as the extraction solvent, so it has the characteristics of being green and protecting the environment [11]. In addition, subcritical water extraction technology is also characterized by high efficiency. For example, a study has shown that the subcritical water extraction method had a higher yield of polysaccharides extracted from lily, compared with the ultrasonic-assisted extraction [12].

Currently, the extraction of tea saponins in subcritical water is mainly focused on the optimization of extraction conditions from *C. oleifera* seed meals and the simultaneous extraction of oil and tea saponins from *C. oleifera* seeds [13,14]. Subcritical water extraction temperature, time, pressure, and the solid–liquid ratio are external factors affecting the extraction rate of tea saponins, while the composition of raw materials is an internal factor affecting the extraction rate of the target substance. For example, there are differences in the polysaccharide yield of different varieties of the *Gastrodia elata Bl* aerial parts under the same extraction conditions [15]. The composition of meals is related to various factors such as varieties and growth environment [16]. Moreover, the extraction conditions of the oil from seeds affect the composition of the meals [17]. *C. oleifera* is widely distributed in southern China, and there are many varieties of *C. oleifera* [18,19]. So, there are differences in the composition of different *C. oleifera* seed meals. At present, there are few studies on the effect of the composition of *C. oleifera* seed meals on the extraction rate of tea saponins under subcritical water extraction conditions.

In this study, we aimed to investigate the effect of the composition of *C. oleifera* seed meals on the extraction rate of tea saponins in subcritical water, to improve the extraction rate of tea saponins. Therefore, the extraction rates of tea saponins from three *C. oleifera* seed meals purchased from Hubei, Hunan, and Guizhou provinces in subcritical water were studied, and the components affecting the extraction of tea saponins were screened. To further explore the mechanism of the influence of the screened components on the extraction rate of tea saponins in subcritical water, the simulated system of commercially available tea saponins, whey protein isolate (WPI), and glucose was reacted under subcritical water conditions, and the mechanism of the effect of WPI and glucose on tea saponin content was analyzed. In order to verify these mechanisms and achieve efficient extraction of tea saponins, alkaline protease was used to change the composition of *C. oleifera* seed meal, and the changes in the extraction rate of tea saponins were analyzed. This paper provides theoretical and technical guidance for the efficient extraction of tea saponins from *C. oleifera* seed meals.

## 2. Materials and Methods

### 2.1. Materials

Tea saponin standard (HPLC ≥ 98%) was provided by Shanghai Ru ji Biotechnology (Shanghai, China). Whey protein isolate (WPI) and alkaline protease were supplied by Shanghai Yuan ye Biotechnology (Shanghai, China). Commercially available tea saponins were purchased from Shanxi Ze lang Biotechnology (Xi’an China), named CA-tea saponins. 8-Anilino-1-naphthalenesulfonic acid (ANS), 3,5-dinitrosalicylic acid, and vanillin were supplied by Macklin (Shanghai, China). Petroleum ether, sulfuric acid, ethyl acetate, anhydrous ethanol, sodium hydroxide, glucose, hydrogen peroxide, phenol, sodium metabisulfite, hydrochloric acid, potassium sodium tartrate, and potassium bromide were purchased from Sinopharm Chemical Reagent (Shanghai, China). All chemicals were of analytical grade unless otherwise stated.

### 2.2. Samples

The three kinds of *C. oleifera* seed meal were purchased from Hubei province, Hunan province, and Guizhou province in China, named HB *C. oleifera* seed meal, HN *C. oleifera* seed meal, and GZ *C. oleifera* seed meal, respectively. The *C. oleifera* seed meals were crushed and passed through a 60-mesh sieve. The residual oil in the *C. oleifera* seed meals was removed with petroleum ether, and the degreased and dried *C. oleifera* seed meals were put into a sealed bag and placed in a desiccator.

### 2.3. Composition of C. oleifera Seed Meals

#### 2.3.1. Determination of Ash, Crude Protein, Crude Fiber, and Amino Acid Content

The determination methods of ash, crude protein, crude fiber, and amino acid content in the *C. oleifera* seed meal were GB 5009.4-2016, 5009.5-2016, 5009.124-2016, GB/T 8310-2013 [20,21,22,23].

#### 2.3.2. Determination of Total Sugar Content

The total sugar extraction procedure from the *C. oleifera* seed meals was determined following the method in [24], with slight modifications, as follows: 0.3 g of *C. oleifera* seed meal was added to a mixture of 15 mL HCl and 50 mL distilled water, then heated at 100 °C for 3 h. After cooling to room temperature, the residue was washed with distilled water, the wash solution was centrifuged, and the supernatant-total sugar solution was collected in a 250 mL volumetric flask. Next, 1 mL of 5% (*w*/*v*) phenol solution and 5 mL of concentrated sulfuric acid were sequentially added to 1 mL of the total sugar solution. The mixture was vortexed thoroughly, left to stand for 10 min, and then incubated in a 30 °C water bath for 20 min. The absorbance of the solution was measured at 490 nm using a UV-1800 spectrophotometer (UV-1800, Shimadzu, Tokyo, Japan). The standard working curve of total sugars was y = 10.987x + 0.05, R^2^ = 0.9956. Based on Equation (1), the total sugar content was quantitatively analyzed, with the results being expressed as grams of total sugars per 100 g of *C. oleifera* seed meal.

(1) $$\;\mathrm{Total}\;\mathrm{sugar}\;\mathrm{content}\;\%=\frac{C_{1}{\cdot V}_{1}\cdot N}{m_{0}}\cdot 100\%$$

where *C*_1_ is the total sugar concentration (mg/mL); *V*_1_ is the volume of the total sugar solution (mL); *N* is the number of dilutions; *m*_0_ is the weight of the *C. oleifera* seed meal (g).

#### 2.3.3. Determination of Reducing Sugar Content

The reducing sugar extraction procedure from the *C. oleifera* seed meals was determined following the method in [25] with slight modifications, as follows: 1 g of *C. oleifera* seed meal was added to 20 mL of distilled water and subjected to ultrasound treatment at 50 °C for 30 min, and centrifuged at 4000 r/min for 20 min. This process was repeated three times, and the supernatant-reducing sugar solution was combined in a 100 mL volumetric flask. Next, 2 mL of 3,5-dinitrosalicylic acid (DNS) reagent was added to 1 mL of the reducing sugar solution, which was heated in a boiling water bath for 15 min. After heating, the solution was cooled to room temperature under running water, and distilled water was added to bring the final volume to 10 mL. The absorbance of the solution was measured at 540 nm using a UV-1800 spectrophotometer (UV-1800, Shimadzu, Tokyo, Japan). The standard working curve of reducing sugars was y = 1.9204x + 0.2026, R^2^ = 0.9948. Based on Equation (2), the reducing sugar content was quantitatively analyzed, with the results being expressed as grams of reducing sugars per 100 g of *C. oleifera* seed meal.

(2) $$\mathrm{Reducing}\;\mathrm{sugar}\;\mathrm{content}\;\%=\frac{C_{2}{\cdot V}_{2}\cdot N}{m_{0}}\cdot 100\%$$

where *C*_2_ is the reducing sugar concentration (mg/mL); *V*_2_ is the volume of reducing sugar solution (mL); *N* is the number of dilutions; *m*_0_ is the weight of the *C. oleifera* seed meal (g).

#### 2.3.4. Determination of Tea Saponin Content

The extraction procedure was as follows: 0.4 g of *C. oleifera* seed meal wrapped in filter paper was subjected to Soxhlet extraction with 80% ethanol in an 80 °C water bath for 5 h, and the ethanol solution–tea saponin solution was combined in a 100 mL volumetric flask. The tea saponin content was determined following the method of Yu et al. [2] with slight modifications: 0.5 mL of 8% (*w*/*v*) vanillin-anhydrous ethanol solution and 4 mL of 77% (*v*/*v*) sulfuric acid solution were sequentially added to 0.5 mL of the tea saponin solution, which was heated in a 60 °C water bath for 15 min. After cooling in an ice water bath for 10 min to reach room temperature, the absorbance of the solution was measured at 550 nm using a UV-1800 spectrophotometer (UV-1800, Shimadzu, Tokyo, Japan). Based on Equation (3), the tea saponins were quantitatively analyzed using a standard calibration curve, y = 1.1694x + 0.1669, R^2^ = 0.9961, with the results being expressed as grams of tea saponins per 100 g of *C. oleifera* seed meal.

(3) $$\;\mathrm{Tea}\;\mathrm{saponin}\;\mathrm{content}\;\left(\%\right)=\frac{C_{3}{\cdot V}_{3}\cdot N}{m_{0}}\cdot 100\%$$

where *C*_3_ is the tea saponin concentration (mg/mL); *V*_3_ is the volume of tea saponin solution (mL); *N* is the number of dilutions; *m*_0_ is the weight of the *C. oleifera* seed meal (g).

### 2.4. Subcritical Water Extraction of Tea Saponins

#### 2.4.1. Determination of Tea Saponin Extraction Rate

The extraction procedure was as follows: 5.0 g of *C. oleifera* seed meal was dissolved in distilled water, stirred evenly, and tea saponins were extracted at 1 MPa, under the following subcritical water extraction conditions: extraction time (20–60 min), solid–liquid ratio (1:18–1:22, *w*/*v*), and temperature (120–160 °C). After extraction, the solution was centrifuged at 4000 r/min for 20 min, and the supernatant was concentrated using a rotary evaporator. Next, 50 mL of 95% ethanol was added to the concentrated solution, which was then filtered. The filtrate was further concentrated, and the rotary flask was rinsed with water to bring the final volume of concentration up to 20 mL. The concentrated solution was added to 20 mL of hydrogen peroxide (30%) and heated in a 60 °C water bath for 60 min. Afterward, the solution was dried at 60 °C in a blast drying oven to obtain a light yellow tea saponin solid powder, and the tea saponin sample was stored in a dryer. The method for determining the tea saponin content was the same as described in Section 2.3.4. Extracted crude tea saponins were dissolved in distilled water, passed through an AB-8 macroporous resin, and eluted sequentially with distilled water followed by 80% ethanol. The eluate was then concentrated and dried to obtain purified tea saponin powder. The purified tea saponin sample was mixed with potassium bromide crystals and scanned using a Fourier transform infrared spectrometer (IS50 Fourier-transform infrared, Suzhou, China) within the spectral range of 400 to 4000 cm^−1^ [2]. Based on Equation (4), the tea saponin extraction rate was quantitatively analyzed using a standard calibration curve, y = 1.1694x + 0.1669, R^2^ = 0.9961, with the results being expressed as grams of tea saponins per 100 g of tea saponins in the *C. oleifera* seed meal.

(4) $$\mathrm{Extraction}\;\mathrm{rate}\;\mathrm{of}\;\mathrm{tea}\;\mathrm{saponins}\;(\%)=\frac{\omega_{1}{\cdot m}_{1}}{m_{0}{\cdot \omega }_{2}}\cdot 100\%$$

where *ω*_1_ is the tea saponins purity of extracted tea saponins (%); *ω*_2_ is the content of tea saponins in the *C. oleifera* seed meal (%); *m*_1_ is the weight of extracted tea saponins (g); *m*_0_ is the weight of the *C. oleifera* seed meal (g).

#### 2.4.2. Determination of Reducing Sugar Dissolution Rate

The crude tea saponin sample extracted from HB, HN, and GZ three *C. oleifera* seed meals was dissolved in distilled water. The content of reducing sugars in the crude tea saponin sample was determined, and the method was the same as described in Section 2.3.3. Based on Equation (5), the reducing sugar dissolution rate was quantitatively analyzed using a standard calibration curve, y = 1.9204x + 0.2026, R^2^ = 0.9948, with the results being expressed as grams of reducing sugars per 100 g of reducing sugars in the *C. oleifera* seed meal.

(5) $$\mathrm{Dissolution}\;\mathrm{rate}\;\mathrm{of}\;\mathrm{reducing}\;\mathrm{sugars}\;(\%)=\frac{\omega_{3}{\cdot m}_{1}}{m_{0}{\cdot \omega }_{4}}\cdot 100\%$$

where *ω*_3_ is the reducing sugar content of the extracted tea saponins (%); *ω*_4_ is the content of the reducing sugars in the *C. oleifera* seed meal (%); *m*_1_ is the weight of the extracted tea saponins (g); *m*_0_ is the weight of the *C. oleifera* seed meal (g).

#### 2.4.3. Determination of Protein Dissolution Rate

The crude tea saponin sample extracted from HB, HN, and GZ three *C. oleifera* seed meals was dissolved in distilled water, and the content of proteins in the crude tea saponin sample was determined, using the Bradford assay [26], with absorbance taken at 595 nm using a Multiskan SkyHigh (Multiskan SkyHigh, Thermo Fisher, Singapore). Based on Equation (6), the protein dissolution rate was quantitatively analyzed using a standard calibration curve, y = 3.6813x + 0.5703, R^2^ = 0.9902, with the results being expressed as grams of proteins per 100 g of proteins in the *C. oleifera* seed meal.

(6) $$\;\mathrm{Dissolution}\;\mathrm{rate}\;\mathrm{of}\;\mathrm{proteins}\;(\%)=\frac{\omega_{5}{\cdot m}_{1}}{m_{0}{\cdot \omega }_{6}}\cdot 100\%$$

where *ω*_5_ is the protein content of extracted tea saponins (%); *ω*_6_ is the content of proteins in the *C. oleifera* seed meal (%); *m*_1_ is the weight of extracted tea saponins (g); *m*_0_ is the weight of the *C. oleifera* seed meal (g).

### 2.5. Changes in CA-Tea Saponin Content in Simulation System

At 120 °C and 1 MPa, the following three factors were considered on the residual rate of CA-tea saponins: (1) the concentration of CA-tea saponins was 3 mg/mL, the mass ratio of WPI and CA-tea saponins was 0.2:3–1.0:3, and the control group was a CA-tea saponin solution at a concentration of 3 mg/mL. (2) In the CA-TS group, the concentration of CA-tea saponins was 3 mg/mL, in the WPI group, the concentration of WPI was 1 mg/mL, and in the WPI+CA-TS group, the mass ratio of WPI and CA-tea saponins was 1.0:3. The reaction time was 20–70 min. (3) The concentration of CA-tea saponins was 1 mg/mL, the mass ratio of glucose and WPI and CA-tea saponins was 1:1:1–5:1:1. In the control group, the concentration of CA-tea saponins was 1 mg/mL and the mass ratio of WPI to CA-tea saponins was 1:1.

CA-tea saponins, WPI, and glucose were dissolved in 100 mL of distilled water according to the above experimental conditions, the pH was adjusted to 4, and the solution was then heated in a reaction kettle at 120 °C and 1 MPa. After heating, the solution was cooled and stored in the refrigerator at 4° C for further use. The method for determining CA-tea saponin content in the solution was the same as described in Section 2.3.4. Based on Equation (7), the CA-tea saponin residual rate was quantitatively analyzed, with the results being expressed as grams of residual CA-tea saponins per 100 g of CA-tea saponins before the reaction.

(7) $$\mathrm{CA}\text{-}\mathrm{tea}\;\mathrm{saponin}\;\mathrm{residual}\;\mathrm{rate}\;(\%)=\frac{C_{4}\cdot N}{C_{5}\cdot W}\cdot 100\%$$

where *C*_4_ is the concentration of the CA-tea saponins after subcritical water heating (mg/mL); *C*_5_ is the concentration of the CA-tea saponins before subcritical water heating (mg/mL); *N* is the number of dilutions; *W* is the purity of CA-tea saponins (%).

### 2.6. Measurement of Browning Degree in Simulation System

Following the method of Tai et al. [27] was used with slight modifications: the reaction solution from Section 2.5, was diluted 16 times, and its absorbance was measured at 294 nm and 420 nm using a UV-1800 spectrophotometer (UV-1800, Shimadzu, Tokyo, Japan).

### 2.7. The Surface Hydrophobicity of WPI in the Simulation System

Surface hydrophobicity is an important indicator that reflects the tertiary structure of proteins [28]. Following the method of Yang et al. [29] with slight modifications, 0.2, 0.4, 0.6, 0.8, and 1.0 mL of the reaction solution from Section 2.5. was added to 5 mL brown test tubes, respectively. Distilled water was added to bring the total volume to 4 mL, followed by the addition of 20 μL (8 mmol/L) of ANS, which was then placed in the dark for 15 min. Fluorescence intensity was measured using an F-4600 spectrometer (F-4600, Hitachi, Tokyo, Japan) at an excitation wavelength of 390 nm and an emission wavelength of 470 nm. The initial slope of fluorescence intensity versus WPI concentration, calculated by linear regression analysis, was used as an indicator of surface hydrophobicity.

### 2.8. Alkaline Protease Enzymatic Hydrolysis of HB C. oleifera Seed Meal

Alkaline protease can break down proteins into small peptides, increasing the content of proteins in the solution [30]. Alkaline protease treatment of the HB *C. oleifera* seed meal can increase the protein content in the solution, which is conducive to the occurrence of the Maillard reaction. The procedure was as follows: 5.0 g of HB *C. oleifera* seed meal was added to 110 mL of distilled water, followed by the alkaline protease dosage of 2%, 3%, 4%, 5% (*w*/*w*) at pH 8 and a 50 °C water bath for 1 h. After enzymatic hydrolysis, the pH of the solution was adjusted to 5.5, and the control group was the HB *C. oleifera* seed meal without alkaline protease treatment. The tea saponins were extracted in subcritical water at 120 °C and 1 MPa for 30 min. The extracted tea saponin sample was obtained according to the experimental method described in Section 2.4.1. The method for determining the tea saponin extraction rate was the same as described in Section 2.4.1.

### 2.9. Statistical Analysis

All assays were performed in triplicate. Data are presented as mean values with standard deviations. Statistical analysis was conducted using Duncan’s test at a significance level of *p* < 0.05, and ANOVA with SPSS 25.0 software.

## 3. Results

### 3.1. Composition of C. oleifera Seed Meals

The contents of the basic components of the HB, HN, and GZ *C. oleifera* seed meals are shown in Table 1. There were differences in the composition of the three *C. oleifera* seed meals. The total sugar content in the HB, HN, and GZ *C. oleifera* seed meals was 28.31%, 24.81%, and 25.62%, and the crude fiber content was 17.06%, 20.91%, and 24.67%, respectively. The content of tea saponins in the HB *C. oleifera* seed meal was the highest, which was 19.30%. The crude protein content in the HB, HN, and GZ *C. oleifera* seed meals was 13.75%, 11.66%, and 10.75%, respectively. The proteins in the HN and GZ *C. oleifera* seed meals consisted of 17 amino acids (Table A1), which agrees with previous studies [31], while the protein in the HB *C. oleifera* seed meal lacked histidine. The ratios of basic amino acids to total amino acids were as follows: HB (12.12%) < HN (15.67%) < GZ (19.06%), and the ratios of non-polar hydrophobic amino acids to total amino acids were as follows: HB (41.93%) > GZ (36.05%) > HN (34.18%).

### 3.2. Extraction Rate of Tea Saponins from HB, HN, and GZ C. oleifera Seed Meals

The extraction rates of tea saponins from the HB, HN, and GZ *C. oleifera* seed meals in subcritical water are shown in Table 2. The extraction rate of tea saponins from the HB *C. oleifera* seed meal decreased from 21.62% to 1.78% as temperature increased from 120 °C to 150 °C. Similarly, the extraction rate of tea saponins from the HN *C. oleifera* seed meal decreased from 29.89% to 4.86% as temperature increased from 120 °C to 160 °C. In contrast, the extraction rate of tea saponins from the GZ *C. oleifera* seed meal initially increased from 17.01% to 22.81% as temperature increased from 120 °C to 130 °C, but then decreased to 1.79% at 160 °C. This is because the plant cell wall was disrupted with increasing temperature [32] to dissolve more tea saponins, whereas the excessive temperature of subcritical water resulted in the degradation of the tea saponins in the solution, which is consistent with previous results reported in the literature [14]. On the other hand, the polarity of water decreases with increasing temperature in subcritical water [33], which may be detrimental to the extraction of tea saponins since tea saponins are polar molecules [34].

At 120 °C and 1 MPa and a solid–liquid ratio of 1:20, the extraction rate of tea saponins from the HN *C. oleifera* seed meal was the highest from 30 min to 60 min compared to the HB and GZ *C. oleifera* seed meals. The extraction rate of tea saponins from the HB and HN *C. oleifera* seed meals increased from 15.42% to 21.62% and 12.30% to 29.89% as the time increased from 20 min to 30 min, respectively. However, the extraction rate of tea saponins decreased to 6.32% and 23.45% at 60 min, respectively. In contrast, the extraction rate of tea saponins from the GZ *C. oleifera* seed meal decreased from 21.13% to 10.29% as the time increased from 20 min to 60 min.

At 120 °C and 1 MPa for 30 min, the extraction rate of tea saponins from the HB and GZ *C. oleifera* seed meals increased from 15.59% to 21.62% and 16.29% to 17.01%, respectively, as the solid–liquid ratio increased from 1:18 to 1:20, at 1:22, the extraction rate of tea saponins decreased to 19.35% and 13.98%, respectively. In contrast, the extraction rate of tea saponins from the HN *C. oleifera* seed meal increased from 25.47% to 31.69% as the solid–liquid ratio increased from 1:18 to 1:22. Under the above subcritical water extraction conditions, the maximum tea saponin extraction rate (21.62%) from the HB *C. oleifera* seed meal was achieved at 120 °C and 1 MPa for 30 min, and a solid–liquid ratio of 1:20; the maximum tea saponin extraction rate (31.69%) from the HN *C. oleifera* seed meal was achieved at 120 °C and 1 MPa for 30 min, and a solid–liquid ratio of 1:22; the maximum tea saponin extraction rate (22.81%) from the GZ *C. oleifera* seed meal was achieved at 130 °C and 1 MPa for 30 min, and a solid–liquid ratio of 1:20.

**Figure 1:**
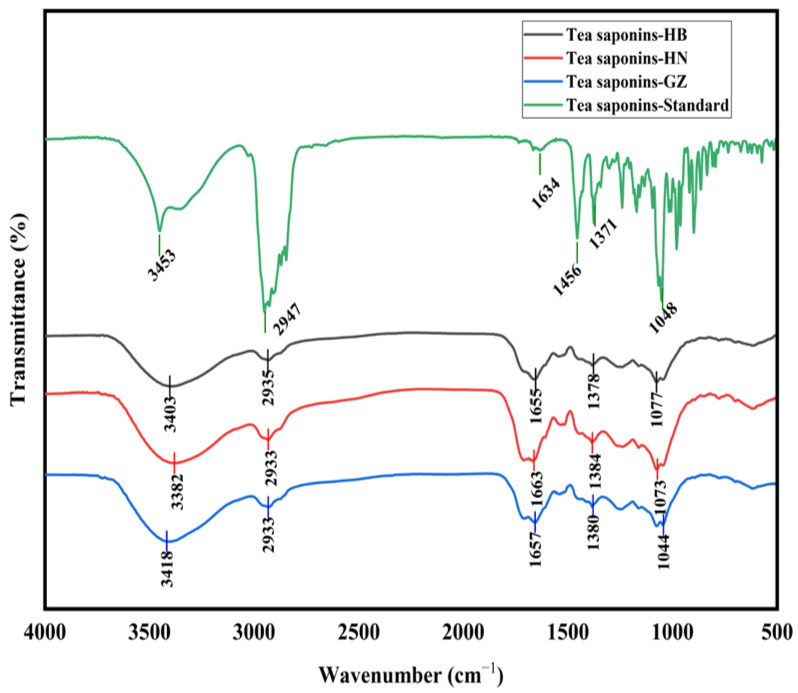
FT-IR spectra of tea saponins extracted from HB, HN, and GZ *C. oleifera* seed meals in subcritical water.

The FT-IR spectra of the tea saponin samples are shown in Figure 1. The tea saponins extracted from the HB, HN, and GZ *C. oleifera* seed meals using subcritical water exhibited the same infrared characteristic peaks as the standard and closely resembled the infrared spectra of tea saponins reported in previous studies [2]. Consequently, these findings confirm that the subcritical water extraction method used in this study successfully extracted tea saponins, which belong to the class of oleanane pentacyclic triterpene saponins.

### 3.3. Dissolution Rates of Reducing Sugars and Proteins from HB, HN, and GZ C. oleifera Seed Meals

The dissolution rates of reducing sugars and proteins from the HB, HN, and GZ *C. oleifera* seed meals are shown in Table 2, at 120 °C and 1 MPa, and a solid–liquid ratio of 1:18–1:22, over 20–60 min. The dissolution rate of proteins from the GZ *C. oleifera* seed meal was relatively high, reaching up to 18.10% at a solid–liquid ratio of 1:20 for 20 min. The dissolution rate of reducing sugars from the HB *C. oleifera* seed meal increased from 27.21% to 39.39% as the time increased from 20 min to 50 min, then decreased to 37.53% at 60 min. The dissolution rate of reducing sugars from the HN *C. oleifera* seed meal increased from 18.96% to 20.49% as the time increased from 20 min to 30 min, decreased to 13.11% at 50 min, and then slowly rose to 15.85% at 60 min. The dissolution rate of reducing sugars from the GZ *C. oleifera* seed meal was 11.99–14.44% (20–60 min). The dissolution rate of reducing sugars was influenced by the composition of the three *C. oleifera* seed meals and the consumption extent of the reducing sugars during subcritical water extraction because reducing sugars can participate in the Maillard reaction with proteins [35]. The order of the dissolution rate of reducing sugars in the three *C. oleifera* seed meals was GZ < HN < HB (20–60 min). The solid–liquid ratio from 1:18 to 1:22 at 120 °C and 1 MPa for 30 min did not significantly affect the dissolution rate of reducing sugars.

Tea saponins contain hydrophilic glycans and hydrophobic saponins [36], which may be less hydrophilic than reducing sugars. In subcritical water, compared with HB and HN *C. oleifera* seed meals, the large dissolution rate of reducing sugars from the HB *C. oleifera* seed meal was not conducive to the extraction of tea saponins. This may be due to the reducing sugars in the HB *C. oleifera* seed meal competing with tea saponins for dissolution. But compared with the GZ and HN *C. oleifera* seed meals, the dissolution rate of reducing sugars from the GZ *C. oleifera* seed meal was lower, which was also not conducive to the extraction of tea saponins. Previous studies have shown that basic amino acids have higher reactivity in the Maillard reaction [37]. Due to the relatively high content of basic amino acids in the GZ *C. oleifera* seed meal (2.35 g/100 g), as shown in Table A1, although the reducing sugar content in the GZ *C. oleifera* seed meal is higher, more reducing sugars can be consumed by the Maillard reaction, which may be the reason for the lower dissolution rate of reducing sugars from the GZ *C. oleifera* seed meal.

Based on the analysis of the experimental results, it can be concluded that the content of reducing sugars and basic amino acids impacted the tea saponin extraction rate in *C. oleifera* seed meals. For the same raw material of *C. oleifera* seed meal, the basic amino acid content can be classified as the protein content, so the content of reducing sugars and proteins in the *C. oleifera* seed meal was the internal factor of the extraction rate of tea saponins in subcritical water.

### 3.4. Changes in CA-Tea Saponins and Maillard-Reaction Products Content in the Simulation System

In order to further study the effects of reducing sugars and proteins on the content of tea saponins in subcritical water, glucose and WPI were selected as the reducing sugar and protein, respectively. Glucose, WPI, and CA-tea saponins composed the simulation system in subcritical water. At 120 °C and 1 MPa for 30 min, the concentration of CA-tea saponins was 3 mg/mL, the residual rate of CA-tea saponins was 92.78%, which was attributed to the hydrolysis of tea saponins in subcritical water.

**Figure 2:**
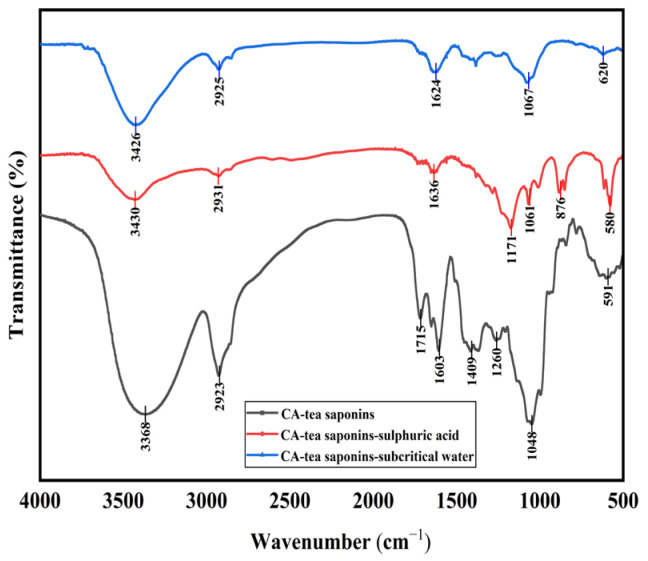
Hydrolysis of CA-tea saponins in subcritical water.

The hydrolysate of CA-tea saponins in subcritical water was consistent with the product catalyzed by sulfuric acid at atmospheric pressure, as confirmed by infrared spectroscopy (Figure 2). Specifically, the intensity of the absorption peaks around 3400 cm^−1^ and 1045 cm^−1^ (ether bond) weakened after the hydrolysis of CA-tea saponins, suggesting the detachment of sugar moieties from the CA-tea saponins in subcritical water. Previous studies have also shown that tea saponins are hydrolyzed into glycogen and saponins in the presence of sulfuric acid under atmospheric pressure [38], and the results of this paper are consistent with their experimental results.

**Figure 3:**
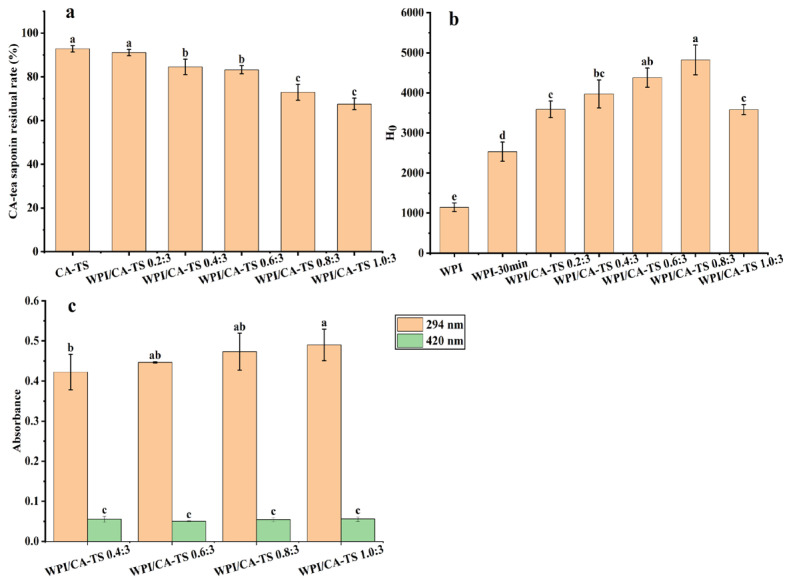
CA-tea saponin residual rate (**a**), the surface hydrophobicity (H_0_) of WPI (**b**), and the content of Maillard-reaction products (**c**) at different mass ratios of WPI and CA-tea saponins. Different lowercase letters indicate significant differences among samples (*p* < 0.05).

Figure 3a. shows that the residual rate of CA-tea saponins gradually decreased from 91.06% to 67.55% when the mass ratio of WPI and CA-tea saponins increased from 0.2:3 to 1.0:3, indicating that WPI can reduce the residual rate of CA-tea saponins in subcritical water.

A study has shown that ginsenosides Re containing glycans can react with alanine under heating conditions, and the content of ginsenosides Re decreased after the Maillard reaction with alanine [39]. Tea saponins contain glycans, and the mixed solution of CA-tea saponins and WPI in subcritical water also produced browning. The absorbance at 294 nm and 420 nm, which indicates the contents of intermediate and final products of the Maillard reaction, respectively [40]. The contents of the Maillard-reaction products in the mixed solution of WPI and CA-tea saponins are shown in Figure 3c. The absorbance increased from 0.422 to 0.490 at 294 nm and remained in the range of 0.05 to 0.056 at 420 nm as the mass ratio of WPI and CA-tea saponins increased from 0.4:3 to 1.0:3. This indicated the involvement of CA-tea saponins in the Maillard reaction with WPI in subcritical water. The degree of the Maillard reaction between WPI and CA-tea saponins increased with the increasing mass ratio of WPI and CA-tea saponins. At this time, the absorbance of the WPI and tea saponin mixed solution at 294 nm and 420 nm with a WPI and CA-tea saponins mass ratio of 0.2:3 could not be used because the solution under this condition appeared cloudy with a light yellow color.

**Figure 4:**
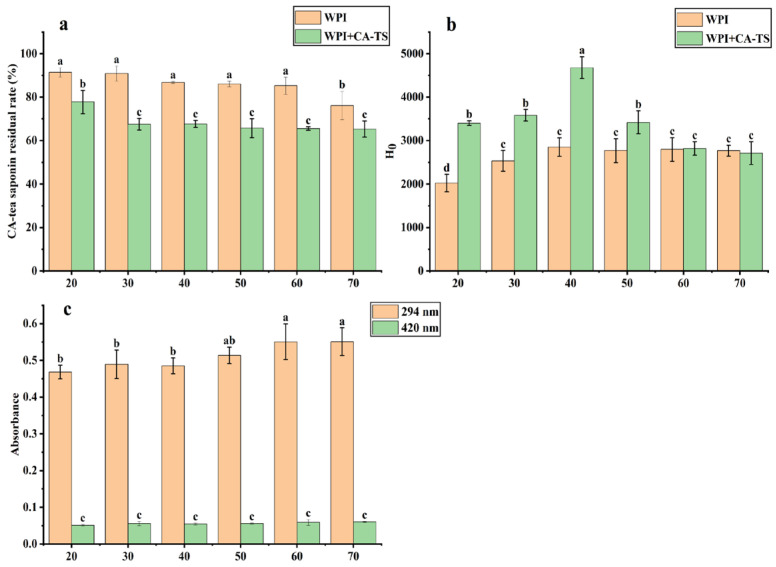
CA-tea saponin residual rate (**a**), the surface hydrophobicity (H_0_) of WPI (**b**), and the content of Maillard-reaction products (**c**) from 20 min to 70 min. Different lowercase letters indicate significant differences among samples (*p* < 0.05).

The effect of the reaction time on the residual rate of CA-tea saponins in subcritical water is shown in Figure 4a. In the CA-TS group, the residual rate of CA-tea saponins was 92.08% at 20 min, decreased from 92.78% to 76.14% as the reaction time increased from 30 min to 70 min, due to the hydrolysis of CA-tea saponins in subcritical water. In contrast, the residual rate of CA-tea saponins decreased from 77.79% to 65.32% over the same time period in the WPI+CA-TS group. The effect of the reaction time on the content of Maillard-reaction products in the WPI and CA-tea saponins mixed solution is shown in Figure 4c. The absorbance increased from 0.468 to 0.551 at 294 nm and from 0.051 to 0.06 at 420 nm as the time increased from 20 min to 70 min, indicating that the content of Maillard-reaction intermediate products by WPI and CA-tea saponins increased with the extension of reaction time.

**Figure 5:**
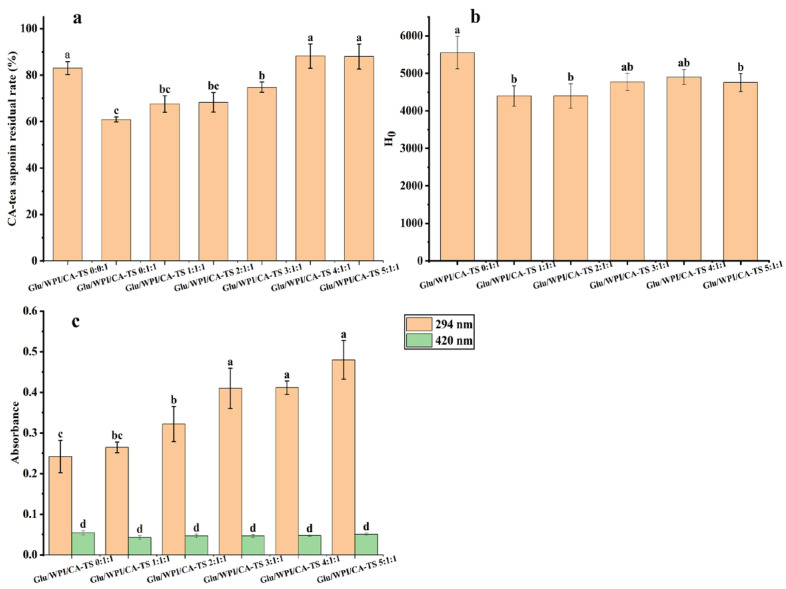
CA-tea saponin residual rate (**a**), the surface hydrophobicity (H_0_) of WPI (**b**), and the content of Maillard-reaction products (**c**) at different mass ratios of glucose and WPI and CA-tea saponins. Different lowercase letters indicate significant differences among samples (*p* < 0.05).

The effect of reducing sugars on the residual rate of CA-tea saponins is shown in Figure 5a. The residual rate of CA-tea saponins increased from 60.87% to 88.19% as the mass ratio of glucose and WPI and CA-tea saponins increased from 0:1:1 to 4:1:1, and was 88.00% at the glucose and WPI and CA-tea saponins mass ratio of 5:1:1. The content of Maillard-reaction products in the glucose, WPI, and CA-tea saponins mixed solution is shown in Figure 5c. The absorbance increased from 0.242 to 0.480 at 294 nm and varied between 0.043 and 0.054 at 420 nm as the mass ratio of glucose and WPI and CA-tea saponins increased from 0:1:1 to 5:1:1, indicating an increase in the content of Maillard-reaction intermediate products. This may be due to the low molecular weight of glucose, which can easily expose the carbonyl group for more efficient and flexible reaction with WPI [41]. However, it had an insignificant effect on the content of the final products (*p* > 0.05). Glucose increased the residual rate of CA-tea saponins and the degree of Maillard reaction, suggesting that glucose may compete with CA-tea saponins to bind to WPI and participate in the Maillard reaction.

### 3.5. Effect of CA-Tea Saponins on the Surface Hydrophobicity of WPI in Subcritical Water

It has been reported in the literature that the surface hydrophobicity of proteins changes when they undergo the Maillard reaction with sugars [42]. To further investigate the mechanism of the Maillard reaction between CA-tea saponins and WPI in subcritical water, the surface hydrophobicity of WPI was investigated.

As shown in Figure 3b, the surface hydrophobicity of WPI increased from 1143.08 to 2533.23 after subcritical water treatment, indicating an exposure of the hydrophobic groups in WPI, which is consistent with previous literature [43]. The surface hydrophobicity of WPI/CA-TS increased from 3587.90 to 4821.17 as the mass ratio of WPI and CA-tea saponins increased from 0.2:3 to 0.8:3, then decreased to 3583.30 at 1.0:3, indicating a further increase in the hydrophobic groups in WPI by the CA-tea saponins as the mass ratio of WPI and CA-tea saponins increased from 0.2:3 to 0.8:3 in subcritical water, in contrast, the decrease in the surface hydrophobicity of WPI/CA-TS at ratio of 1.0:3 was attributed to the Maillard reaction between WPI and CA-tea saponins, formed more WPI-sugar combinations, which is also consistent with the literature [44].

The effect of reaction time on the surface hydrophobicity of WPI is shown in Figure 4b. In the WPI group, the surface hydrophobicity of WPI increased from 2022.70 to 2852.27 as the time increased from 20 min to 40 min and remained stable from 50 min to 70 min. This is because the structural disruption of the protein increased, exposing more hydrophobic groups as the heating time increased [45]. In the WPI+CA-TS group, the surface hydrophobicity of WPI/CA-TS increased from 3401.47 to 4679.33 as the time increased from 20 min to 40 min, then decreased to 2711.30 at 70 min. This trend can be explained by the increased structural disruption of the proteins and exposure of more hydrophobic amino acids as the reaction time increased from 20 min to 40 min, whereas when the reaction time increased from 50 min to 70 min, more WPI underwent a Maillard reaction with the CA-tea saponins and the reducing sugars in them, resulting in a decrease in the surface hydrophobicity of the WPI/CA-TS, which is in agreement with the literature [46].

The effect of glucose on the surface hydrophobicity of WPI is shown in Figure 5b. The surface hydrophobicity of WPI/CA-TS/Glucose was reduced after adding glucose, due to the introduction of -OH groups to WPI by glucose, which is also consistent with previous studies [47].

### 3.6. Validation of the Extraction Mechanism of Tea Saponins

**Figure 6:**
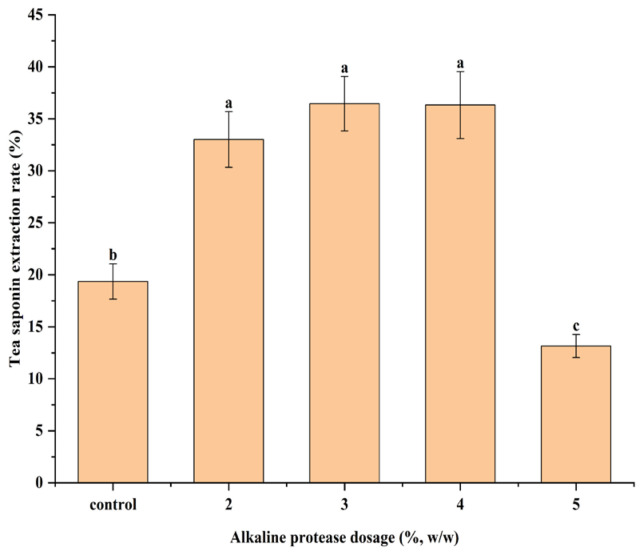
Effect of alkaline protease on tea saponin extraction rate. Different lowercase letters indicate significant differences among samples (*p* < 0.05). The control group was not enzymatically hydrolyzed HB *C. oleifera* seed meal.

To validate the effect of the reducing sugars and proteins on tea saponin extraction rate and improve the extraction rate of tea saponins, alkaline protease was used to regulate the content of reducing sugars and proteins in the HB *C. oleifera* seed meal. Alkaline proteases can break down proteins into small-molecule peptides, increasing the degree of protein hydrolysis and exposing more free amino groups [48]. The results are shown in Figure 6. The tea saponin extraction rate increased from 33.01% to 36.45% at the alkaline protease dosage of 2% to 3%, and decreased to 13.15% at the alkaline protease dosage of 5%. The tea saponin extraction rate was 19.35% in the control group.

Compared to the control group, the increase in the tea saponin extraction rate at an alkaline protease dosage of 2% to 3% may be attributed to the reduced amount of reducing sugars, which inhibited the competitive dissolution of reducing sugars. This occurred as the alkaline protease increases protein dissolution, consuming more reducing sugars by participating in a Maillard reaction with proteins in subcritical water. The extraction rate of tea saponins decreased at the alkaline protease dosage of 5%, which may be because the reducing sugars were consumed in large quantities and cannot effectively inhibit the Maillard reaction of tea saponins with proteins.

## 4. Conclusions

At 120 °C, 1 MPa, and a solid–liquid ratio of 1:20, the tea saponin extraction rate from the HN *C. oleifera* seed meal was the highest as the time increased from 30 min to 60 min, among the three HB, HN, and GZ *C. oleifera* seed meals, the maximum tea saponin extraction rate (31.69%) was achieved at 120 °C and 1 MPa for 30 min, and a solid–liquid ratio of 1:22. By comparing the extraction rates of saponins, and the dissolution rates of reducing sugars and proteins in three *C. oleifera* seed meals, it was found that the content of reducing sugars and proteins can affect the extraction rate of tea saponins. The dissolution rate of reducing sugars in the HB *C. oleifera* seed meal was high, so the reduced sugar content in the HB *C. oleifera* seed meal is relatively high in subcritical water, which was not conducive to the extraction of tea saponins. The simulation system showed that CA-tea saponins can be hydrolyzed, and the residual rate of CA-tea saponins decreased. WPI can further reduce the content of CA-tea saponins through the Maillard reaction, and the mechanism of action of WPI is to synergize the degradation of CA-tea saponins. Glucose can inhibit the Maillard reaction between CA-tea saponins and WPI, and the mechanism of action of glucose reverses the protection of CA-tea saponins. The quantitative relationship of these mechanisms is influenced by the reaction conditions. At 120 °C and 1 MPa for 30 min, the CA-tea saponin concentration of 1 mg/mL, the WPI and CA-tea saponins mass ratio of 1:1, the content of CA-tea saponins decreased by 39.13%, of which the hydrolysis of CA-tea saponins accounted for 16.97%, and the Maillard reaction between WPI and CA-tea saponins accounted for 22.16%. The residual rate of CA-tea saponins increased by 44.88% at a glucose to WPI to CA-tea saponins mass ratio of 4:1:1.

After alkaline protease treatment, the content of reducing sugars and proteins in the HB *C. oleifera* seed meal was changed, and the extraction rate of tea saponins was affected. The extraction rate of tea saponins increased by 88.37% at an alkaline protease dosage of 3%, compared with the control group. These provide theoretical guidance for the efficient extraction of tea saponins from *C. oleifera* seed meals. In this study, only three kinds of *C. oleifera* seed meal were selected as experimental materials, which had certain limitations, and the raw materials suitable for tea saponin extraction could be screened out by selecting a variety of experimental materials in a later study.

## Figures and Tables

**Table 1:** Composition of *C. oleifera* seed meals.

Composition (%)	HB *C. oleifera* Seed Meal	HN *C. oleifera* Seed Meal	GZ *C. oleifera* Seed Meal
Total sugars	28.31 ± 0.77 ^a^	24.81 ± 0.33 ^b^	25.62 ± 1.45 ^b^
Reducing sugars	11.36 ± 0.58 ^b^	9.38 ± 0.55 ^c^	15.12 ± 1.05 ^a^
Ash	4.44 ± 0.16 ^b^	4.87 ± 0.05 ^a^	3.60 ± 0.17 ^c^
Crude fibers	17.06 ± 1.74 ^c^	20.91 ± 0.15 ^b^	24.67 ± 0.73 ^a^
Crude proteins	13.75 ± 0.47 ^a^	11.66 ± 0.12 ^b^	10.75 ± 0.86 ^b^
Tea saponins	19.30 ± 0.74	16.64 ± 1.43	16.56 ± 0.63

Different lowercase letters (a–c) indicate significant differences (*p* < 0.05) by Duncan’s test within the same row.

**Table 2:** Tea saponin extraction rate, and reducing sugar and protein dissolution rates.

Solid–Liquid Ratio	Temperature (°C)	Time (min)	Tea Saponin Extraction Rate (%)			Reducing Sugar Dissolution Rate (%)			Protein Dissolution Rate (%)		
			HB	HN	GZ	HB	HN	GZ	HB	HN	GZ
1:20	120	30	21.62 ± 1.60 ^a^	29.89 ± 2.73 ^a,b^	17.01 ± 1.31 ^b^						
1:20	130	30	19.33 ± 1.44 ^a,b^	23.87 ± 1.83 ^c^	22.81 ± 0.32 ^a^						
1:20	140	30	13.64 ± 0.10 ^d^	21.69 ± 1.98 ^c^	12.14 ± 0.29 ^d,e^						
1:20	150	30	1.78 ± 0.18 ^g^	6.62 ± 0.82 ^e^	6.76 ± 0.55 ^f^						
1:20	160	30	2.30 ± 0.33 ^g^	4.86 ± 0.90 ^e^	1.79 ± 0.28 ^g^						
1:20	120	20	15.42 ± 1.29 ^c,d^	12.30 ± 0.63 ^d^	21.13 ± 2.18 ^a^	27.21 ± 1.01 ^e^	18.96 ± 1.68 ^a,b^	13.78 ± 1.17	12.21 ± 1.25 ^b^	16.25 ± 0.88 ^a^	18.10 ± 0.22 ^a^
1:20	120	30	21.62 ± 1.60 ^a^	29.89 ± 2.73 ^a,b^	17.01 ± 1.31 ^b^	31.47 ± 0.89 ^c,d,e^	20.49 ± 2.02 ^a,b^	14.44 ± 1.41	14.03 ± 0.89 ^a^	12.22 ± 1.05 ^c,d^	14.79 ± 0.83 ^b,c^
1:20	120	40	17.47 ± 1.43 ^b,c^	25.78 ± 2.19 ^b,c^	16.19 ± 1.57 ^b,c^	34.97 ± 3.27 ^a,b,c^	17.66 ± 0.65 ^b,c^	11.99 ± 0.87	11.79 ± 1.27 ^b^	11.56 ± 1.01 ^c,d^	14.06 ± 0.67 ^b,c^
1:20	120	50	11.21 ± 1.08 ^e^	25.13 ± 2.50 ^c^	16.59 ± 1.08 ^b^	39.39 ± 3.58 ^a^	13.11 ± 1.23 ^d^	13.03 ± 1.04	10.61 ± 0.28 ^b^	13.56 ± 1.09 ^b,c^	13.91 ± 1.25 ^b,c^
1:20	120	60	6.32 ± 0.21 ^f^	23.45 ± 0.56 ^c^	10.29 ± 0.88 ^e^	37.53 ± 2.66 ^a,b^	15.85 ± 0.75 ^c,d^	13.11 ± 1.04	11.84 ± 0.76 ^b^	11.04 ± 0.50 ^d^	13.34 ± 1.26 ^c^
1:18	120	30	15.59 ± 0.58 ^c,d^	25.47 ± 1.68 ^c^	16.29 ± 0.95 ^b,c^	29.15 ± 1.64 ^d,e^	19.14 ± 1.11 ^a,b^	13.15 ± 1.20	10.31 ± 0.26 ^b^	14.56 ± 1.03 ^a,b^	16.35 ± 1.41 ^a,b^
1:20	120	30	21.62 ± 1.60 ^a^	29.89 ± 2.73 ^a,b^	17.01 ± 1.31 ^b^	31.47 ± 0.89 ^c,d,e^	20.49 ± 2.02 ^a,b^	14.44 ± 1.41	14.03 ± 0.89 ^a^	12.22 ± 1.05 ^c,d^	14.79 ± 0.83 ^b,c^
1:22	120	30	19.35 ± 1.69 ^a,b^	31.69 ± 1.19 ^a^	13.98 ± 1.28 ^c,d^	32.75 ± 2.97 ^b,c,d^	21.24 ± 0.17 ^a^	12.40 ± 1.19	11.85 ± 0.98 ^b^	15.12 ± 1.05 ^a,b^	15.01 ± 1.37 ^b,c^

Different lowercase letters (a–g) indicate significant differences (*p* < 0.05) by Duncan’s test within the same column.

## Data Availability

The original contributions presented in this study are included in the article. Further inquiries can be directed to the corresponding author.

## Abbreviations

WPI	Whey protein isolate
TS	Tea saponin
CA-tea saponin	Commercially available tea saponins
HB *C. oleifera* seed meal	HuBei *C. oleifera* seed meal
HN *C. oleifera* seed meal	HuNan *C. oleifera* seed meal
GZ *C. oleifera* seed meal	GuiZhou *C. oleifera* seed meal

## Appendix A

**Table A1:** Amino acid composition of three kinds of *C. oleifera* seed meal.

Types of Amino Acids	HB (g/100 g)	HN (g/100 g)	GZ (g/100 g)
Glu	2.64 ± 0.10	3.16 ± 0.00	2.62 ± 0.00
Asp	1.20 ± 0.05	1.39 ± 0.00	1.11 ± 0.01
Thr	0.55 ± 0.02	0.59 ± 0.00	0.47 ± 0.00
Ser	0.64 ± 0.02	0.71 ± 0.01	0.55 ± 0.01
Pro	0.49 ± 0.02	0.59 ± 0.01	0.51 ± 0.02
Gly	0.68 ± 0.01	0.74 ± 0.00	0.58 ± 0.01
Cys	0.65 ± 0.00	0.66 ± 0.02	0.50 ± 0.01
Val	0.63 ± 0.00	0.65 ± 0.00	0.53 ± 0.00
Ala	0.84 ± 0.02	0.89 ± 0.01	0.76 ± 0.01
Leu	1.13 ± 0.03	1.27 ± 0.01	1.11 ± 0.02
Met	0.19 ± 0.01	0.22 ± 0.00	0.08 ± 0.00
Ile	0.47 ± 0.00	0.51 ± 0.00	0.45 ± 0.02
Tyr	0.78 ± 0.01	0.25 ± 0.00	0.22 ± 0.01
Phe	1.83 ± 0.08	0.63 ± 0.00	0.52 ± 0.01
Lys	0.67 ± 0.01	0.60 ± 0.01	0.72 ± 0.02
His	n.d.	0.35 ± 0.00	0.31 ± 0.01
Arg	1.08 ± 0.02	1.33 ± 0.00	1.33 ± 0.02
Total	14.44 ± 0.00	14.52 ± 0.00	12.33 ± 0.05
alkaline amino acids	1.75 ± 0.01	2.28 ± 0.01	2.35 ± 0.02
non-polar hydrophobic amino acids	3.83 ± 0.05	4.55 ± 0.00	3.73 ± 0.01
non-polar hydrophobic amino acids	6.06 ± 0.01	4.96 ± 0.01	4.45 ± 0.01

Note: n.d. indicates not detected.
